# Vitamin D status in a multi-ethnic population of northern Norway: the SAMINOR 2 Clinical Survey

**DOI:** 10.1017/S1368980018003816

**Published:** 2020-05

**Authors:** Natalia Petrenya, Christel Lamberg-Allardt, Marita Melhus, Ann Ragnhild Broderstad, Magritt Brustad

**Affiliations:** 1 Department of Community Medicine, Faculty of Health Sciences, UiT The Arctic University of Norway, Postboks 6050 Langnes 9037, Tromsø, Norway; 2 Calcium Research Unit, Department of Food and Nutrition Sciences, University of Helsinki, Helsinki, Finland; 3 Centre for Sami Health Research, Department of Community Medicine, UiT The Arctic University of Norway, Tromsø, Norway

**Keywords:** Vitamin D status, Serum 25-hydroxyvitamin D concentration, Predictors, Intake of vitamin D, Indigenous population Sami, Norway

## Abstract

**Objective:**

To investigate serum 25-hydroxyvitamin D (S-25(OH)D) concentration in a multi-ethnic population of northern Norway and determine predictors of S-25(OH)D, including Sami ethnicity.

**Design:**

Cross-sectional data from the second survey of the Population-based Study on Health and Living Conditions in Regions with Sami and Norwegian Populations (the SAMINOR 2 Clinical Survey, 2012–2014). S-25(OH)D was measured by the IDS-iSYS 25-Hydroxy Vitamin Dˢ assay. Daily dietary intake was assessed using an FFQ. BMI was calculated using weight and height measurements.

**Setting:**

Ten municipalities of northern Norway (latitude 68°–70°N).

**Participants:**

Males (*n* 2041) and females (*n* 2424) aged 40–69 years.

**Results:**

Mean S-25(OH)D in the study sample was 64·0 nmol/l and median vitamin D intake was 10·3 µg/d. The prevalence of S-25(OH)D<30 nmol/l was 1·9 % and <50 nmol/l was 24·7 %. In sex-specific multivariable linear regression models, older age, blood sample collection in September–October, solarium use, sunbathing holiday, higher alcohol intake (in females), use of cod-liver oil/fish oil supplements, use of vitamin/mineral supplements and higher intakes of vitamin D were significantly associated with higher S-25(OH)D, whereas being a current smoker and obesity were associated with lower S-25(OH)D. These factors explained 21–23 % of the variation in S-25(OH)D.

**Conclusions:**

There were many modifiable risk factors related to S-25(OH)D, however no clear ethnic differences were found. Even in winter, the low prevalence of vitamin D deficiency found among participants with non-Sami, multi-ethnic Sami and Sami self-perceived ethnicity was likely due to adequate vitamin D intake.

Vitamin D regulates Ca and P homeostasis and is essential for the health of bones, teeth and muscles^(^^1^^)^. People obtain vitamin D through UVB-activated skin production and through dietary intake^(^^2^^)^; however, natural dietary sources of vitamin D are limited. In the Norwegian diet, the most valuable sources include cod-liver oil supplements, oily fish, fortified butter and margarine, in addition to traditional foods like cod liver and hard roe^(^^3^^–^^5^^)^.

Currently, 25-hydroxyvitamin D (25(OH)D) concentration is the best indicator of vitamin D status. There is an ongoing debate on the optimal concentration of 25(OH)D^(^^6^^)^. In terms of bone health, the US Institute of Medicine has stated that, for 97·5 % of the world’s population, a 25(OH)D concentration of 50 nmol/l would be equivalent to vitamin D sufficiency; a concentration of <30 nmol/l is attributed to a risk of vitamin D deficiency^(^^7^^)^.

Hypovitaminosis D is prevalent in the European population. A recent publication on vitamin D deficiency in Europe found that 40·4 and 13·0 % of tested individuals had 25(OH)D concentration <50 nmol/l and <30 nmol/l, respectively^(^^8^^)^.

Several population-based studies have evaluated the distribution of and factors related to 25(OH)D concentration in the Norwegian population^(^^4^^,^^9^^–^^14^^)^. The Vitamin D Standardization Program (VDSP)^(^^15^^,^^16^^)^ created a protocol that can be used for retrospective standardization of 25(OH)D data to make them comparable with other standardized studies. It was applied to two nationally representative surveys of adults aged 30 years and 45 years or older in Norway. The results showed a lower prevalence of 25(OH)D<50 nmol/l and <30 nmol/l compared with other European studies: 15·0 and 1·3 %, respectively, in a cohort from Oslo (the HUBRO Study, Norwegians with Pakistan ethnicity were excluded)^(^^17^^)^; and 18·6 and 0·9 %, respectively, in a cohort from Tromsø (the Tromsø Study–6th Survey)^(^^8^^)^. Seasonal fluctuation in 25(OH)D concentration has been well documented in European populations^(^^8^^)^, and studies from Norway have also reported drops in 25(OH)D concentration during winter^(^^4^^,^^11^^,^^13^^)^. However, because vitamin D supplements like cod-liver oil are widely used in Norway^(^^18^^)^, the amplitude of seasonal fluctuations in 25(OH)D in northern Norway is smaller than in central European countries^(^^19^^)^.

Population groups that may be at high risk of vitamin D deficiency in Norway include elderly nursing home residents, particularly those who do not use vitamin D supplements^(^^20^^)^, adolescents^(^^9^^)^ and certain non-Western immigrant groups^(^^12^^,^^21^^,^^22^^)^. In fact, in the HUBRO Study, 65 % of Norwegians with Pakistan ethnicity had a 25(OH)D concentration of <30 nmol/l and 92 % had a concentration of <50 nmol/l^(^^17^^)^.

In the Norwegian population, predictors of 25(OH)D concentration include daily dietary intake of vitamin D^(^^4^^)^, intake of vitamin D supplements^(^^11^^,^^13^^)^ and season^(^^4^^,^^11^^,^^13^^,^^23^^)^. In addition, 25(OH)D concentration has been found to be positively associated with increased physical activity^(^^11^^,^^13^^)^, frequent alcohol consumption^(^^13^^)^ and estimated daily hours of exposure to UVB radiation^(^^4^^)^. On the other hand, 25(OH)D concentration has been negatively associated with current smoking^(^^13^^)^, high BMI^(^^11^^,^^13^^)^ and not having a holiday at southern latitudes during the previous summer^(^^4^^)^.

Vitamin D status is of special concern in northern geographical areas. In the most northern part of Norway (69–71°N), UVB-activated skin production of vitamin D is limited or absent for a considerable part of the year (October–March)^(^^24^^)^. Fish liver and fresh fish-liver oil, which have some of the highest vitamin D content, are traditionally consumed in northern Norway, but mainly in coastal communities^(^^5^^)^. Cod (*Gadus mothua* L.) liver is consumed during the winter months (January–March/April) and saithe (*Pollachius virens*) liver is consumed from late summer until autumn (July–September/October). Reduction of consumption of traditional foods has been shown to be associated with insufficient nutrient intakes and a decrease in circulating 25(OH)D concentration among certain Indigenous populations in the Arctic^(^^25^^,^^26^^)^.

The population of northern Norway is multi-ethnic and comprises the Indigenous ethnic group Sami. Nutritional rickets already received attention in this population in 1928–1929, when a high prevalence of this condition was reported among children in those Sami communities in Finnmark, where there is limited access to seafood^(^^27^^)^. A dietary pattern analysis based on the first survey of the Population-based Study on Health and Living Conditions in Regions with Sami and Norwegian Populations (the SAMINOR 1 Survey, 2003–2004) showed that dietary patterns differed by geographical region and ethnicity^(^^28^^)^. A dietary pattern that included a high intake of reindeer meat was dominant in inland areas and among participants with a strong Sami affiliation, while higher fish consumption was common in coastal areas. To the best of our knowledge, no large-scale mixed-gender studies on vitamin D status that cover both inland and coastal geographical areas of northern Norway have been conducted. Furthermore, no studies have explored this issue according to Sami ethnicity. The main goal of the present study was to determine serum 25(OH)D (S-25(OH)D) concentration in a multi-ethnic population of northern Norway and to investigate predictors of vitamin D status, including Sami ethnicity.

## Methods

### Study design and population

**Fig. 1:**
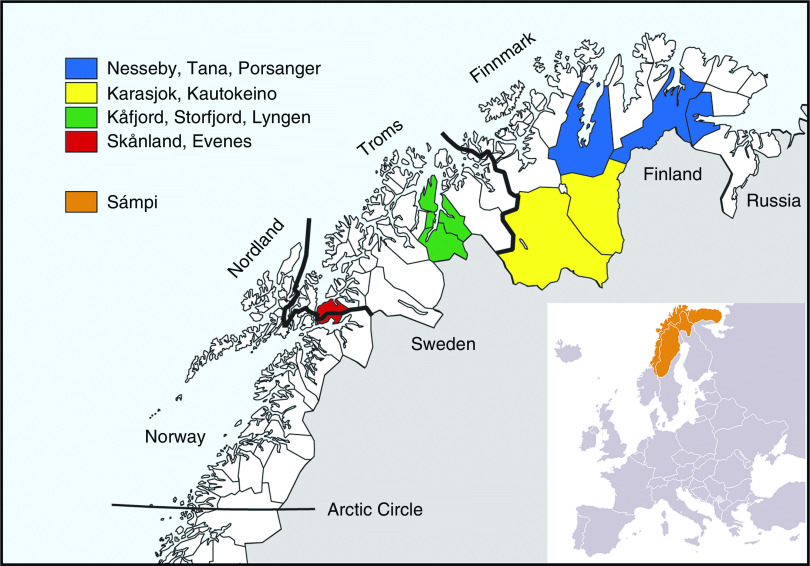
Map of study sites

The second survey of the Population-based Study on Health and Living Conditions in Regions with Sami and Norwegian Populations (the SAMINOR 2 Clinical Survey) was conducted by the Centre for Sami Health Research, UiT The Arctic University of Norway. Data collection was carried out in ten municipalities of northern Norway (Skånland, Evenes, Kåfjord, Storfjord, Karasjok, Kautokeino, Porsanger, Tana, Nesseby and Lyngen; latitude 68–70°N) in 2012–2014 (Fig. 1). These municipalities were selected based on a large proportion of Sami inhabitants. All inhabitants aged 40–79 years and residing in these municipalities were invited to participate in the study by personal letter. Data were collected through an eight-page self-administered questionnaire (see www.saminor.no for an English translation of the questionnaire), a short clinical examination, and blood sample collection and analysis. The questionnaire was prepared in Norwegian and then translated into the Northern Sami language. The questionnaire distributed to participants aged 40–69 years (*n* 10 399) contained a four-page FFQ. In this age group, 4876 attended the survey (participation rate 47 %). The response was higher among females (54 %) than males (40 %).

### Determination of serum 25-hydroxyvitamin D concentration

Non-fasting blood samples were collected during the clinical examination. Sampling in Skånland and Evenes was performed in September–October 2012, in Karasjok in January–February 2013, in Kautokeino in February–March 2013, in Porsanger in April–May 2013, in Kåfjord in September–October 2013, in Storfjord in October–November 2013, in Tana and Nesseby in February–April 2014, and in Lyngen in May–June 2014.

No sampling was performed in December, July or August. Resultant serum samples were stored at –70°C and used to assess S-25(OH)D concentration by the IDS-iSYS 25-Hydroxy Vitamin Dˢ assay on the IDS-iSYS analyser (IDS Ltd, Boldon, UK) at the Department of Food and Environmental Sciences, University of Helsinki (*n* 4832). The assay is validated and certified by the VDSP. Reproducibility was ensured by adhering to the Vitamin D External Quality Assessment Scheme (DEQAS; http://www.deqas.kpmd.co.uk). The inter-assay and intra-assay CV were 4·7 and 8·0 %, respectively.

### Questionnaire data

#### Ethnicity

Ethnicity was determined on the basis of self-perception and was assessed by the following question: ‘What do you consider yourself to be?’ Response options were (i) ‘Norwegian’, (ii) ‘Sami’, (iii) ‘Kven’ and (iv) ‘Other; if other, please describe’. Multiple answers were allowed. Based on these answers, we generated three ethnic categories: non-Sami, multi-ethnic Sami and Sami self-perceived ethnicity. The non-Sami group included participants who considered themselves as something other than Sami; that is, Norwegian, Kven, other, or a combination of these. The multi-ethnic Sami group included participants who defined themselves as Sami in combination with any other ethnic group. The Sami group included participants who defined themselves solely as Sami; that is, without reporting any other ethnicities. We excluded participants with backgrounds from non-Western European, Asian and African countries.

#### Food and nutrient assessment

The FFQ in the SAMINOR 2 Clinical Survey was a slightly modified version of that used in the Norwegian Women and Cancer (NOWAC) Study^(^^29^^)^, with minor adjustments, mainly related to some known traditional food items^(^^30^^)^. The NOWAC FFQ has previously been validated for the general female population of Norway and is described in detail elsewhere^(^^30^^,^^31^^)^. We utilized the NOWAC nutrient calculation program to estimate daily intakes of foods and nutrients, and the Norwegian food composition database (see www.matportalen.no/verktoy/the_norwegian_food_composition_table/) was used. Total daily dietary intake of vitamin D (µg/d) was calculated. Liquid cod-liver oil supplement (translated as ‘bottled cod-liver oil’ in the English version of the questionnaire, question nos. 88–90; see www.saminor.no) was estimated in g/d and included in the calculation of daily dietary vitamin D intake; however, other supplements were not.

#### Use of dietary supplements

*Frequency of use of cod-liver oil/fish oil supplements*. We considered both liquid cod-liver oil and cod-liver oil capsules/fish oil capsules as cod-liver oil/fish oil supplements. Participants were asked to report their intake of these supplements during winter and during the rest of the year. Participants who took such supplements daily throughout the year were defined as ‘whole-year daily users’. Participants who took cod-liver oil/fish oil supplements daily either during winter or the rest of the year were defined as ‘part-year daily users’. Participants who took cod-liver oil/fish oil supplements between one and six days per week either during winter or the rest of the year were defined as ‘occasional users’. Participants who never took cod-liver oil/fish oil supplements or took it less frequently than once per week (during winter and the rest of the year) were defined as ‘non-users’.

*Frequency of use of cod-liver oil capsules/fish oil capsules*. We created the variable ‘frequency of use of cod-liver oil capsules/fish oil capsules’ for use in analyses that included total daily dietary vitamin D intake, as liquid cod-liver oil was already included therein. In this way, we could examine the contribution of this factor alone. Participants who took cod-liver oil capsules/fish oil capsules daily during winter and during the rest of the year were defined as ‘whole-year daily users’. Participants who took cod-liver oil capsules/fish oil capsules daily either during winter or the rest of the year were defined as ‘part-year daily users’. Participants who never took cod-liver oil capsules/fish oil capsules or took it less frequently than once per day (during winter and the rest of the year) were defined as cod-liver oil capsules/fish oil capsules ‘non-users’.

#### Use of vitamins/minerals

Participants were asked: ‘Do you take food supplements (vitamins/minerals)?’ Use of vitamin/mineral supplements other than cod-liver oil/fish oil supplements (yes or no) was estimated and used in the analyses as a dichotomous categorical variable. It was not possible to include vitamins/minerals and cod-liver oil capsules/fish oil capsules into estimation of daily vitamin D intake due to lack of information on nutrient content in the various products used.

#### Non-dietary variables

The questionnaire included questions on education (total number of completed years of education), physical activity (on a scale from 1 to 10) and smoking status. In the analyses, education was categorized as <13 years and ≥13 years; physical activity as low (1–3), moderate (4–7) and high (8–10); and smoking status as current, former and never.

Sun exposure was estimated by questions on sunbathing holidays in the past month (yes or no), frequency of solarium use in the past month (no use, 1–2 times per week, ≥3 times per week) and total number of daylight hours outside in the past week. In the analyses, total number of daylight hours outside in the past week was adjusted for the ‘dark season’ when the intensity of sunlight is insufficient for cutaneous vitamin D production in this geographical area^(^^24^^)^ (both missing and reported values were coded as 0 if blood samples were collected between 1 November and 15 February), divided into sex-specific quartiles and used as a categorical variable.

### Height and weight

Height and weight were measured using an electronic Height, Weight & Fatness Measuring System (DS-103; Dongsahn Jenix, Seoul, Korea) with the participants wearing light clothing and no shoes. Height was measured to the nearest 0·1 cm and weight to the nearest 100 g; and these were then used to calculate BMI in kg/m^2^. Obesity was defined as BMI≥30·0 kg/m^2^.

### Register-based variables

Gender, year of birth and municipality of residence was obtained from the National Register (Folkeregisteret). Age was defined as the age at the end of the year of clinical examination and was divided into three groups: 40–49, 50–59 and 60–69 years. Geographical region of residence was categorized as the inland region (including the municipalities of Karasjok and Kautokeino) and the coastal region (including the other eight municipalities), based on whether the municipalities include coastal areas or not (Fig. 1).

### Seasons

We divided the sample into three groups according to season of blood sample collection: (i) November, January–April (extended Arctic winter); (ii) May–June; and (iii) September–October.

### Exclusions

We excluded participants with missing information on self-perceived ethnicity (*n* 115), non-Western migrants (*n* 69), those with incomplete FFQ (more than 50 % blanks, i.e. more than fifty-seven food items without responses; *n* 91), those with missing height and weight measurements (*n* 7), over- and under-reporters (participants in the top and bottom 1 % of the ratio of energy intake to BMR; *n* 90), those with missing s-25(OH)D concentration (*n* 37) and those with extreme s-25(OH)D concentration (i.e. >200 nmol/l; *n* 2). Totally, 8 % (*n* 411) of the participants were excluded, thus the final analytical sample consisted of 4465 individuals. The minimum–maximum values of total daily energy intake after exclusion were 3·1–22·1MJ in males and 2·6–15·1MJ in females.

### Statistical analysis

We present: (i) mean (sd) S-25(OH)D concentration in males and females by month of blood sample collection; (ii) proportions of males and females with S-25(OH)D concentration <50 nmol/l by month of blood sample collection; (iii) summary statistics of S-25(OH)D concentrations and the proportion of participants with concentration <25, <30, <50 and <75 nmol/l for the entire sample, by gender and season of blood sample collection; and (iv) mean (sd) S-25(OH)D concentration in males and females by characteristics of the study population. We also show mean (sd) and median S-25(OH)D concentrations in non-Sami, multi-ethnic Sami and Sami ethnic groups by season of blood sample collection and age (see online supplementary material, Supplemental Table S1).

S-25(OH)D concentration cut-offs were chosen based on current definitions of vitamin D deficiency (US Institute of Medicine and other experts)^(^^7^^,^^32^^,^^33^^)^. According to the US Institute of Medicine, persons are at risk of deficiency in terms of the bone health at a S-25(OH)D concentration of <30 nmol/l; not all persons, but some, are potentially at the risk of inadequacy at a concentration of 30–<50 nmol/l; practically all persons are vitamin D sufficient at a S-25(OH)D concentration of ≥50 nmol/l^(^^7^^)^. In Europe it is common to define severe deficiency at S-25(OH)D concentration of <25 nmol/l^(^^33^^)^. The Endocrine Society in the USA has suggested that to maintain the best effect of vitamin D on metabolism, S-25(OH)D concentration should be ≥75 nmol/l; however, this is still being debated^(^^32^^)^.

Gender-specific tertiles of total daily dietary vitamin D intake (including liquid cod-liver oil) were created, with the lowest tertile used as the reference group.

First, we applied gender-specific multiple linear regression to assess predictors for S-25(OH)D concentration. Preliminary models included known or suspected determinants of these concentrations, namely age, education, ethnicity, geographical region of residence, smoking status, physical activity, BMI, alcohol intake, seasons, number of daylight hours outside in the past week, sunbathing holiday in the past month, solarium use in the past month, use of vitamins/minerals, use of cod-liver oil capsules/fish oil capsules (because liquid cod-liver oil was included in daily vitamin D intake calculation) and daily vitamin D intake. Backward selection was performed to identify predictors (*P*<0·05). If at least one category was significantly associated with S-25(OH)D concentration, the categorical variable was kept in the final model. The assumptions of the linear regression models were met.

For the final models, the variables age, smoking status, alcohol intake (females), BMI, seasons, sunbathing holiday in the past month, solarium use in the past month, use of vitamins/minerals, use of cod-liver oil capsules/fish oil capsules and daily vitamin D intake were identified as predictors of vitamin D status.

We present models that consider vitamin D intake as a categorical variable (tertiles) and as a continuous variable to estimate the effect of dietary vitamin D intake (µg/d) on S-25(OH)D concentration. Unstandardized *β* coefficient with 95 % CI, *P* value and adjusted *R*^2^ are reported for multivariable linear regression models.

*R*^2^ statistics were employed for dominance analysis to explore the relative importance of predictors (%). General dominance statistic and rank are reported in the online supplementary material, Supplemental Table S2.

Second, we analysed the effect of ethnicity (multi-ethnic Sami *v*. non-Sami (reference) and Sami *v*. non-Sami (reference)) on the probability of having S-25(OH)D concentration <50 or ≥50 nmol/l by multivariable logistic models. One model adjusted for age group and month of blood draw; and the other adjusted for age group, month of blood draw, sunbathing holiday, food supplements other than cod-liver oil and vitamin D intake (strong modifiable predictors in males and females). OR with 95 % CI and *P* value are reported for logistic regression models.

*P*<0·05 was considered statistically significant and all statistical tests were two-sided. Data were analysed using the statistical software package Stata version 14.

## Results

### Distribution of serum 25-hydroxyvitamin D concentration and sample characteristics

**Fig. 2:**
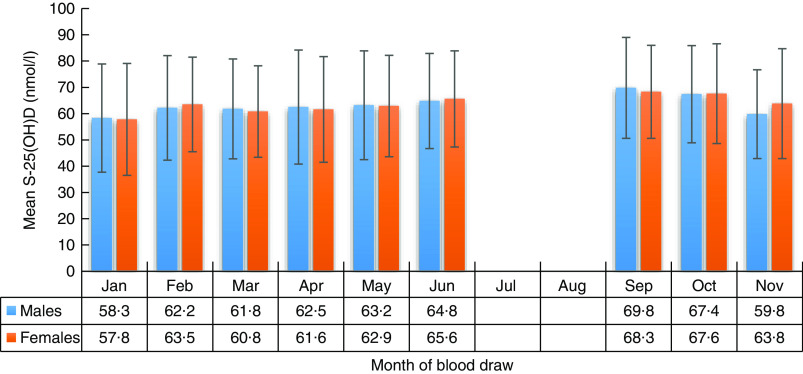
Serum 25-hydroxyvitamin D (S-25(OH)D) concentration (nmol/l) in males and females, by month of blood draw, in the SAMINOR 2 Clinical Survey of adults aged 40–69 years in northern Norway (68–70°N), 2012–2014 (*n* 4465). Values are means with their sd indicated by vertical bars

**Fig. 3:**
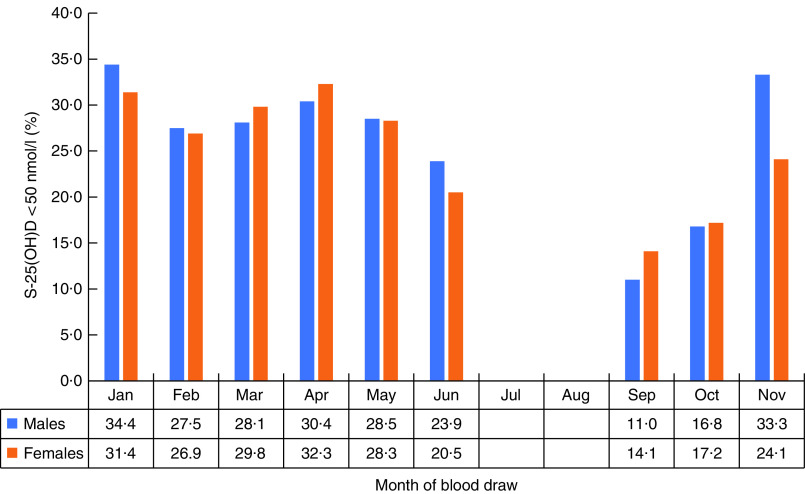
Percentage with 25-hydroxyvitamin D (S-25(OH)D) concentration<50 nmol/l in males and females, by month of blood draw, in the SAMINOR 2 Clinical Survey of adults aged 40–69 years in northern Norway (68–70°N), 2012–2014 (*n* 4465)

The distribution of S-25(OH)D concentration varied by month of blood sample collection in males and females (Figs 2 and 3). The lowest mean S-25(OH)D values and percentage of concentrations <50 nmol/l were detected in January and the highest in September. The mean S-25(OH)D concentration was 64·0 (sd 19·2) nmol/l, with no differences (*P=*0·7) between males (64·1 (sd 19·7) nmol/l) and females (63·9 (sd 18·8) nmol/l; Table 1).

**Table 1:** Distribution of serum 25-hydroxyvitamin D (S-25(OH)D) concentration (nmol/l) in relation to season and gender in the SAMINOR 2 Clinical Survey of adults aged 40–69 years in Northern Norway (68–70°N), 2012–2014 (*n* 4465)

	S-25(OH)D (nmol/l)					
	Entire sample	November, January–April	May–June	September–October	Males	Females
*n*	4465	2165	985	1315	2041	2424
Mean	64·0	61·8	63·4	67·9	64·1	63·9
sd	19·2	19·0	19·7	18·6	19·7	18·8
Minimum	12·8	12·8	16·8	16·4	15·9	12·8
Maximum	183·2	168·7	183·2	164·2	183·2	159·6
5th percentile	35·9	35·1	35·1	40·2	35·8	36·2
25th percentile	50·2	48·0	48·6	55·1	50·4	50·0
Median	62·4	60·4	61·7	66·0	61·9	62·8
75th percentile	75·7	73·3	75·9	79·1	75·5	75·8
95th percentile	97·7	95·7	97·7	100·8	98·7	96·7
<25 nmol/l (%)	0·7	0·9	0·5	0·4	0·6	0·7
<30 nmol/l (%)	1·9	2·2	2·3	0·9	1·8	1·9
<50 nmol/l (%)	24·7	29·0	27·4	15·7	24·5	25·0
<75 nmol/l (%)	74·2	78·2	73·6	68·1	74·4	74·1

The mean age in the study sample population was 55·9 (sd 8·5) years, and females were slightly younger (*P*=0·0001). About half of all participants consumed cod-liver oil/fish oil supplements occasionally or daily. Only 18·2 % of males reported use of vitamins/minerals (other than cod-liver oil/fish oil supplements) compared with 42·3 % of females (*P*<0·0001). There were fewer teetotallers and a higher alcohol intake among males than among females (*P*<0·0001). In the entire sample, 11·9 % had been on a sunbathing holiday and 4·6 % had used a solarium during the past month. Females reported solarium use more often (*P*<0·0001) than males; however, an equal percentage of males and females had been on a sunbathing holiday (~12 %; *P*=0·85). Males reported more daylight hours outside (*P*<0·0001; Table 2).

**Table 2:** Mean serum 25-hydroxyvitamin D (S-25(OH)D) concentration (nmol/l), by sample characteristics, in the SAMINOR 2 Clinical Survey of adults aged 40–69 years in Northern Norway (68–70°N), 2012–2014 (*n* 4465)*

	Males (*n* 2041)				Females (*n* 2424)				
	Characteristic		S-25(OH)D (nmol/l)		Characteristic		S-25(OH)D (nmol/l)		
	Mean, sd or *n*	Median or %	Mean	sd	Mean, sd or *n*	Median or %	Mean	sd	*P* †
Age (years)	56·5	58·0			55·5	56·0			0·0001
sd	8·4				8·6				
Age (years), *n* and %									
40–49	515	25·2	58·0	18·3	686	28·3	57·7	18·0	0·005
50–59	647	31·7	64·3	19·7	806	33·3	64·4	18·1	
60–69	879	43·1	67·5	19·7	932	38·4	68·0	18·7	
Education (years)	12·2	12·0			13·2	13·0			<0·0001
sd	3·5				3·9				
Education (years), *n* and %									
<13	1156	58·4	64·1	20·1	1113	47·6	64·8	19·1	<0·0001
≥13	824	41·6	64·2	19·3	1223	52·4	63·1	18·4	
Self-perceived ethnicity, *n* and %									
Non-Sami	1238	60·7	64·9	20·2	1480	61·1	65·3	19·1	0·95
Multi-ethnic Sami	285	14·0	64·0	19·6	332	13·7	62·1	18·3	
Sami	518	25·4	62·2	18·6	612	25·2	61·6	18·1	
Geographical region, *n* and %									
Inland	429	21·0	60·0	17·5	563	23·2	61·1	17·3	0·08
Coastal	1612	79·0	65·2	20·1	1861	76·8	64·8	19·1	
Smoking status, *n* and %									
Never	715	35·2	63·7	18·5	878	36·7	64·3	18·1	<0·0001
Former	944	46·5	66·1	20·2	978	40·8	65·8	19·0	
Current	370	18·2	59·9	20·1	539	22·5	59·6	18·7	
Physical activity (levels), *n* and %									
Low (1–3)	424	21·1	62·4	21·2	414	17·7	61·5	18·7	<0·0001
Moderate (4–7)	1287	64·2	63·9	19·2	1491	63·7	64·2	18·8	
High (8–10)	295	14·7	67·4	19·2	436	18·6	65·2	19·0	
BMI (kg/m^2^)	28·4	27·9			27·8	26·9			<0·0001
sd	4·1				4·9				
BMI (kg/m^2^), *n* and %									
<30	1444	70·7	65·6	19·9	1744	71·9	64·8	19·3	0·38
≥30, obese	597	29·3	60·5	19·0	680	28·1	61·5	17·3	
Alcohol intake, non-consumers included (g/d)	5·3	2·7			2·8	1·3			<0·0001§
sd	7·1				4·0				
Alcohol intake (g/d), *n* and %									
0, non-consumers	354	17·3	60·9	17·6	635	26·2	61·6	17·5	<0·0001
<6	1102	54·0	64·3	20·0	1466	60·5	64·2	18·7	
≥6	585	28·7	65·5	20·3	323	13·3	67·2	20·9	
Season of the blood collection, *n* and %									
November, January–April	970	47·5	61·8	19·7	1195	49·3	61·8	18·4	0·46
May–June	454	22·2	63·5	20·3	531	21·9	63·3	19·1	
September–October	617	30·2	68·1	18·7	698	28·8	67·8	18·6	
Daylight hours outside in the past week	20·8	15·0			10·3	7·0			<0·0001§
sd	19·4				10·8				
Daylight hours outside in the past week, *n* and %									
Quartile 1	476	25·3	62·4	19·3	624	29·5	60·9	18·2	
Quartile 2	512	27·3	64·6	20·7	469	22·2	64·0	17·7	
Quartile 3	479	25·5	64·4	19·0	526	24·9	65·5	18·7	
Quartile 4	411	21·9	66·1	19·4	494	23·4	65·1	19·3	
Sunbathing holiday in the past month, *n* and %									
Yes	244	12·0	77·0	23·0	285	11·8	74·0	18·5	0·85
No	1784	88·0	62·3	18·6	2121	88·2	62·5	18·4	
Solarium use in the past month, *n* and %									
No	1974	97·6	63·8	19·7	2239	93·6	63·3	18·7	<0·0001
1–2 times	33	1·6	73·1	17·2	116	4·9	72·0	17·9	
≥3 times	15	0·7	79·4	20·3	36	1·5	76·3	16·8	
Use of vitamin/mineral supplements, *n* and %									
Users	360	18·2	72·0	20·1	993	42·3	68·4	18·9	<0·0001
Non-users	1622	81·8	62·2	19·1	1353	57·7	60·8	18·1	
Cod-liver oil/fish oil supplements, *n* and %									
Non-users	1064	52·3	59·1	19·0	1077	44·6	58·9	18·3	<0·0001
Occasional users,≥1/week	256	12·6	64·1	18·6	277	11·5	61·2	17·3	
Part-year daily users	233	11·4	70·3	19·4	402	16·7	67·3	17·0	
Whole-year daily users	482	23·7	72·3	18·5	657	27·2	71·0	18·6	
Cod-liver oil capsules/fish oil capsules, *n* and %									
Non-users/occasional users,<1/d	1419	79·1	64·1	20·0	1500	68·8	63·3	19·1	<0·0001
Part-year daily users	113	6·3	66·4	18·3	244	11·2	63·5	16·7	
Whole-year daily users	262	14·6	69·5	18·7	436	20·0	68·5	18·4	
Vitamin D intake (µg/d)	14·7	11·6			12·1	9·2			<0·0001§
sd	9·5				8·7				
Vitamin D intake (µg/d), *n* and %									
Tertile 1	681	33·4	58·6	19·6	808	33·3	59·5	18·8	
Tertile 2‡	680	33·3	62·5	18·7	808	33·3	62·5	17·8	
Tertile 3‡	680	33·3	71·1	18·7	808	33·3	69·7	18·2	

* Subgroups may not total 4465 due to missing values.

† Differences between males and females were tested by Pearson’s *χ* ^2^ test or the independent-samples *t* test for normally distributed variables.

‡ Cut-off points between tertile 1 and tertile 2 are 9·1 µg/d for males and 7·1 µg/d for females; cut-off points between tertile 2 and tertile 3 are 15·8 µg/d for males and 12·3 µg/d for females.

§ Mann–Whitney *U* test for non-normal data.

The median total daily dietary intake of vitamin D (including liquid cod-liver oil) was 10·3 µg/d for the entire sample, 11·6 µg/d among males and 9·2 µg/d among females (Table 2).

Excluded participants did not differ from the study sample in terms of mean concentration of S-25(OH)D and prevalence of deficiency/insufficiency; however, a somewhat higher prevalence of S-25(OH)D deficiency (4·4 %) and insufficiency (35·5 %) was observed among participants in top and bottom 1 % of energy intake:BMR (*n* 90).

### Distribution of ethnic groups according to season

In November and January–April, 46·4 % of participants with Sami, 16·5 % with multi-ethnic Sami and 37·1 % with non-Sami self-perceived ethnicity were examined. Fewer participants with Sami (5·7 and 5·3 %) and multi-ethnic Sami (11·0 and 11·5 %) than non-Sami ethnicity (83·3 and 83·2 %) were examined in May–June and September–October, respectively (*P*<0·0001).

### Predictors of serum 25-hydroxyvitamin D concentration in males and females

Gender-specific multivariable regression models explained 21–23 % of the variance in circulating S-25(OH)D concentration. Ethnicity did not predict S-25(OH)D concentration in multivariable models. Older age, blood draw in September–October, solarium use in the past month, being on a sunbathing holiday in the past month, higher alcohol intake (in females), more frequent cod-liver capsules/fish oil capsules use, use of vitamin/mineral supplements and higher daily intake of vitamin D were significantly associated with higher S-25(OH)D concentration, whereas being a current smoker and obesity were associated with lower S-25(OH)D concentration in males and females (models 1, 2, 3 and 4 in Table 3).

**Table 3:** Predictors of serum 25-hydroxyvitamin D (S-25(OH)D) concentration (nmol/l), by gender, in the SAMINOR 2 Clinical Survey of adults aged 40–69 years in Northern Norway (68–70°N), 2012–2014 (*n* 4465)

	Males						Females					
	Model 1*			Model 2†			Model 3‡			Model 4§		
	*β*	95 % CI	*P*	*β*	95 % CI	*P*	*β*	95 % CI	*P*	*β*	95 % CI	*P*
Age (years)												
40–49	Ref.		–	Ref.		–	Ref.		–	Ref.		–
50–59	4·39	2·14, 6·65	0·0001	4·51	2·27, 6·75	0·0001	6·28	4·42, 8·14	<0·0001	6·31	4·47, 8·14	<0·0001
60–69	6·07	3·91, 8·23	<0·0001	6·16	4·02, 8·31	<0·0001	9·24	7·40, 11·08	<0·0001	9·03	7·21, 10·85	<0·0001
Smoking status												
Never	Ref.		–	Ref.		–	Ref.		–	Ref.		–
Current	−2·92	−5·36, −0·48	0·02	−2·91	−5·34, −0·49	0·02	−4·16	−6·15, −2·17	<0·0001	−4·11	−6·08, −2·15	<0·0001
Former	1·45	−0·43, 3·33	0·13	1·55	−0·33, 3·42	0·11	0·77	−0·90, 2·43	0·37	0·77	−0·87, 2·42	0·36
BMI (kg/m^2^)												
<30	Ref.		–	Ref.		–	Ref.		–	Ref.		–
≥30, obese	−4·24	−6·11, −2·37	<0·0001	−4·47	−6·33, −2·61	<0·0001	−3·94	−5·57, −2·30	<0·0001	−3·91	−5·53, −2·28	<0·0001
Alcohol intake (g/d)												
0, non-consumers	–	–	–	–	–	–	Ref.		–	Ref.		–
<6	–	–	–	–	–	–	1·44	−0·32, 3·19	0·11	1·64	−0·09, 3·38	0·06
≥6	–	–	–	–	–	–	3·75	1·27, 6·23	0·003	3·63	1·18, 6·08	0·004
Season of blood collection												
November, January–April	Ref.		–	Ref.		–	Ref.		–	Ref.		–
May–June	0·63	−1·54, 2·81	0·57	0·60	−1·56, 2·77	0·59	−0·02	−1·91, 1·87	0·99	−0·004	−1·87, 1·87	1·0
September–October	4·80	2·84, 6·75	<0·0001	4·80	2·86, 6·75	<0·0001	3·64	1·92, 5·36	<0·0001	3·57	1·87, 5·27	<0·0001
Sunbathing holiday in the past month												
No	Ref.		–	Ref.		–	Ref.		–	Ref.		–
Yes	14·79	12·23, 17·35	<0·0001	15·16	12·60, 17·71	<0·0001	9·93	7·68, 12·19	<0·0001	9·90	7·67, 12·13	<0·0001
Solarium use in the past month												
No	Ref.		–	Ref.		–	Ref.		–	Ref.		–
1–2 times	11·70	4·96, 18·43	0·0007	12·69	5·99, 19·39	0·0002	10·23	6·92, 13·53	<0·0001	10·36	7·08, 13·63	<0·0001
≥3 times	14·94	5·60, 24·28	0·002	15·58	6·28, 24·87	0·001	9·62	3·71, 15·53	0·001	10·21	4·36, 16·06	0·0006
Vitamin/mineral supplements												
Users	Ref.		–	Ref.		–	Ref.		–	Ref.		–
Non-users	−5·76	7·96, −3·57	<0·0001	−5·51	−7·70, −3·32	<0·0001	−5·54	−7·07, −4·02	<0·0001	−5·33	−6·83, −3·82	<0·0001
Cod-liver oil capsules/fish oil capsules												
Non-users/occasional users,<1/d	Ref.		–	Ref.		–	Ref.		–	Ref.		–
Part-year daily users	0·32	−3·16, 3·79	0·86	0·25	−3·20, 3·71	0·87	0·26	−2·12, 2·64	0·83	0·86	−1·51, 3·23	0·48
Whole-year daily users	3·60	1·12, 6·08	0·004	3·77	1·30, 6·24	0·003	2·81	0·89, 4·73	0·004	3·10	1·20, 5·00	0·001
Vitamin D intake (µg/d)												
Tertile 1	Ref.		–	–	–	–	Ref.		–	–	–	–
Tertile 2	4·04	1·93, 6·14	<0·0001	–	–	–	1·99	0·19, 3·80	0·03	–	–	–
Tertile 3	10·76	8·69, 12·83	<0·0001	–	–	–	8·87	7·07, 10·68	<0·0001	–	–	–
Vitamin D intake (µg/d)				0·49	0·40, 0·57	<0·0001				0·51	0·43, 0·59	<0·0001
Constant	71·89			69·73			64·83			61·86		
Observations	1719			1719			2065			2065		
Adjusted *R* ^2^	0·21			0·22			0·21			0·23		

Ref., reference category.

* Model 1 in males predictors include: age (categorical), smoking status (categorical), BMI (categorical), seasons (categorical), sunbathing holiday (categorical), solarium use (categorical), food supplements other than cod-liver oil (categorical), use of cod-liver oil capsules/fish oil capsules (categorical) and vitamin D intake (categorical).

† Model 2 in males predictors include: age (categorical), smoking status (categorical), BMI (categorical), season (categorical), sunbathing holiday (categorical), solarium use (categorical), food supplements other than cod-liver oil (categorical), use of cod-liver oil capsules/fish oil capsules (categorical) and vitamin D intake (continuous).

‡ Model 3 in females predictors include: age (categorical), smoking status (categorical), BMI (categorical), season (categorical), sunbathing holiday (categorical), solarium use (categorical), alcohol intake (categorical), food supplements other than cod-liver oil (categorical), use of cod-liver oil capsules/fish oil capsules (categorical) and vitamin D intake (categorical).

§ Model 4 in females predictors include: age (categorical), smoking status (categorical), BMI (categorical), season (categorical), sunbathing holiday (categorical), solarium use (categorical), alcohol intake (categorical), food supplements other than cod-liver oil (categorical), use of cod-liver oil capsules/fish oil capsules (categorical) and vitamin D intake (continuous).

According to the final multivariable regression models, for every additional 1 µg/d (40IU/d) increase in dietary vitamin D intake (including liquid cod-liver oil intake), S-25(OH)D increased by 0·49 nmol/l in males and by 0·51 nmol/l in females (models 2 and 4 in Table 3).

### Multivariable logistic regression models in males and females to assess ethnic differences in the prevalence of serum 25-hydroxyvitamin D<50 nmol/l

In our sample, S-25(OH)D concentration<50 nmol/l was detected in 22·8 % of males and 22·3 % females with non-Sami, 26·7 % males and 27·7 % females with multi-ethnic, and in 27·2 % males and 29·9 % females with Sami self-perceived ethnicity (Table 4). Ethnicity was not associated with S-25(OH)D concentration in males. After adjustment for age and month of blood sample collection, females with Sami self-perceived ethnicity had 30 % reduced odds to have sufficient S-25(OH)D concentration (≥50 nmol/l) compared with non-Sami females (model 1 in Table 4). However, after adjustment for additional variables (age group, month of blood draw, sunbathing holiday in the past month, use of supplements other than cod-liver oil and vitamin D intake), the association was no longer significant (model 2 in Table 4).

**Table 4:** Serum 25-hydroxyvitamin D (S-25(OH)D) concentration (nmol/l) and vitamin D intake (µg/d), by gender and self-perceived ethnicity, in the SAMINOR 2 Clinical Survey of adults aged 40–69 years in Northern Norway (68–70°N), 2012–2014 (*n* 4465)

	Males												Females											
		S-25(OH)D (nmol/l)					Logistic regression models							S-25(OH)D (nmol/l)					Logistic regression models					
	Vitamin D intake (µg/d)		<30 nmol/l		<50 nmol/l		Model 1*			Model 2†			Vitamin D intake (µg/d)		<30 nmol/l		<50 nmol/l		Model 1*			Model 2†		
Entire sample/Jan–Nov	Median	Median	*n*	%	*n*	%	OR	95 %CI	*P*	OR	95 % CI	*P*	Median	Median	*n*	%	*n*	%	OR	95 % CI	*P*	OR	95 % CI	*P*
Non-Sami	11·3	62·2	20	1·6	282	22·8	Ref.		–	Ref.		–	9·1	64·4	32	2·2	330	22·3	Ref.		–	Ref.		–
Multi-ethnic Sami	13·1	62·9	8	2·8	76	26·7	0·9	0·7, 1·2	0·5	0·8	0·5, 1·1	0·1	9·2	60·3	5	1·5	92	27·7	0·9	0·7, 1·2	0·5	0·9	0·6, 1·2	0·4
Sami	11·1	60·4	9	1·7	141	27·2	0·9	0·7, 1·2	0·6	1·0	0·7, 1·3	0·8	9·4	60·1	9	1·5	183	29·9	0·7	0·6, 1·0	0·03	0·8	0·6, 1·1	0·1
Total	11·6	61·9	37	1·8	499	24·5	–	–	–	–	–	–	9·2	62·8	46	1·9	605	25·0	–	–	–	–	–	–

Ref., reference category.

* Model 1: in logistic models with dependent variable S-25(OH)D concentration<50 *v*.≥50 nmol/l, effect of ethnicity was adjusted for age and month of blood draw.

† Model 2: in logistic models with dependent variable S-25(OH)D concentration<50 *v*.≥50 nmol/l, effect of ethnicity was adjusted for age, month of blood draw, sunbathing holiday, food supplements other than cod-liver oil and vitamin D intake (categorical).

## Discussion

The present study investigates vitamin D status in a large multi-ethnic cohort of 4465 individuals aged 40–69 years, including 25 % of participants with self-perceived Sami ethnicity and 14 % of participants with self-perceived multi-ethnic ethnicity from a comprehensive rural area of northern Norway. The results show that, overall, the proportion of participants with non-optimal vitamin D concentration (i.e. <50 nmol/l) was nearly 25 %; however, only 2 % had a vitamin D concentration<30 nmol/l (i.e. severe hypovitaminosis D). Among all predictors, dietary vitamin D intake (including liquid cod-liver oil) and having gone on a sunbathing holiday in the past month were the strongest contributors to maintain optimal vitamin D status in males and females.

Very few individuals had S-25(OH)D concentration <30 nmol/l in all ethnic groups, and this was a striking finding. In fact, this finding is consistent with previous results reported in Nordic countries among populations living close to the Arctic Circle^(^^34^^)^. We found that the population of rural northern Norway has a more favourable vitamin D status than mid-European populations despite limited sun exposure^(^^8^^,^^19^^)^. The factors behind this low prevalence of vitamin D deficiency are likely to include significantly higher dietary vitamin D intake and frequent vitamin D supplementation. However, other factors like paler skin pigmentation in Northerners^(^^35^^)^, sun holidays and different sun-seeking behaviours, and genetic polymorphism could also influence vitamin D status.

Circulating 25(OH)D in the Indigenous Sami in Norway has not been previously studied. We showed no ethnic differences in the prevalence of S-25(OH)D concentration≥50 nmol/l in males. Sami females, compared with non-Sami females, had a 30 % reduced chance of having an optimal S-25(OH)D concentration of ≥50nmol/l after adjustment for age and month of blood sample collection. However, this weak association disappeared when modifiable lifestyle covariates were included in the regression model.

It should be mentioned that sampling in inland areas, where the majority of the population is Sami (Karasjok and Kautokeino), was performed in January, February and March. During these months, UV-induced vitamin D production is limited or absent, leading to a drop in S-25(OH)D concentration, following well-known seasonal fluctuation. Indeed, blood sample collection in September–October was positively associated with higher S-25(OH)D concentration, and the prevalence of concentration<50 nmol/l was lower in September–October compared with November–June. Thus, hypovitaminosis D might be overestimated in our Sami participants. In the present study, blood samples were not collected in July and August, when sun exposure is at its highest; therefore we might have overestimated the prevalence of insufficient S-25(OH)D in the entire sample on average in the year, and in the summer season as well. Seasonal variation was, however, small compared with that reported in Central Europe, and it was in line with data from northern Norway^(^^19^^,^^36^^)^.

Due to nutrition transition, especially among the younger generations, certain aboriginal populations living in the Arctic have a high risk of poor vitamin D status^(^^37^^)^. Data from Greenland (the Inuit Health in Transition study) demonstrated a considerable decrease in S-25(OH)D concentration from 1987 to 2005–2010 (in both periods blood was drawn in May–June)^(^^38^^)^, with an average S-25(OH)D in 2005–2010 of 34 nmol/l and 33 nmol/l among 18–29-year-old males and females, respectively, and 50 nmol/l among the 50–69-year-old males and females. In the present study, average S-25(OH)D concentrations of Sami males and females (62 nmol/l), multi-ethnic Sami males (64 nmol/l) and multi-ethnic Sami females (62 nmol/l) were satisfactory.

Vitamin D status was associated with intake of daily dietary vitamin D in a previous paper on factors associated with 25(OH)D in Norwegian women^(^^4^^)^ and thus we expected that this could also be an important factor in our study. We included ethnicity in the present study due to its assumed influence on dietary traditions and habits. However, the identification of ethnic groups in multi-ethnic population-based studies is challenging, as there are no standardized and validated methods. Diet and dietary traditions are often described as important carriers, markers and tools for cultural and ethnic identity and belonging. Therefore, we found it relevant to categorize ethnicity based on self-perception. We distinguished between participants who considered themselves solely as Sami and those who considered themselves as Sami in addition to other ethnic groups. This allowed us to create two Sami groups that had different dietary habits^(^^39^^)^. We also tested an ethnicity variable that was previously used to analyse data from the SAMINOR 1 Survey^(^^40^^)^. In the present study, Sami ethnicity was determined based on both subjective (Sami self-perception) and objective (Sami language connection and Sami ethnic background) criteria. This variable was not selected as a predictor for vitamin D status in the final linear regression models; thus results from regression models did not differ (data not shown).

Different laboratory methods to assess 25(OH)D concentration have been used in population-based surveys and have led to substantial variability^(^^8^^)^, with original results often deviating considerably from standardized results^(^^15^^,^^17^^)^. We used automated chemiluminescent immunoassay IDS-iSYS 25-Hydroxy Vitamin D^S^ to assess S-25(OH)D concentrations. This method has been validated and certified by the VDSP. However, automated chemiluminescent immunoassays tend to overestimate circulating 25(OH)D concentrations^(^^41^^)^.

In our study, age was positively associated with S-25(OH)D concentration and this association was consistent across all the ethnic groups (Supplemental Table S1). These associations have been previously reported in middle-aged (44–59 years) Norwegian females living at 65–71°N^(^^4^^)^. However, age was not correlated with 25(OH)D concentration in a study of younger (19–55 years old) Norwegian males and females from Nord-Trøndelag (the HUNT Study) living at 64°N with a higher prevalence of hypovitaminosis D (40 % had concentration <50 nmol/l on average during four seasons, with greater prevalence in winter 60 % than in summer 20 %)^(^^13^^)^. In the paper from mid-Norway, daily vitamin D intake was not measured^(^^13^^)^. One explanation for the higher S-25(OH)D in older adults in our study is the more frequent consumption of supplements on a daily basis and a higher total daily vitamin D intake (data not shown). In fact, higher frequency of cod-liver oil/fish oil supplement use was strongly associated with satisfactory vitamin D status. Older individuals may eat more traditional foods, such as oily fish and cod liver, during winter. In the present study, the mean S-25(OH)D concentration was ≥50 nmol/l in the winter months (November, January–April) in males and females (62 nmol/l). It is important to note that our study was performed in the rural part of northern Norway. A previous study showed that 25(OH)D concentrations in a rural coastal settlement of northern Norway in March were high (mean 67·2 nmol/l, 15·4 % individuals had a concentration<50 nmol/l)^(^^5^^)^, and this was explained by high consumption of cod liver and fresh cod-liver oil during extended Arctic winter among this population.

In the present study, dietary vitamin D intake was adequate. Overall, median dietary vitamin D intake was 11 µg/d in Sami and non-Sami males, 13 µg/d in multi-ethnic Sami males and 9 µg/d in Sami, multi-ethnic Sami and non-Sami females. Indeed, studies from Norway that measure both vitamin D intake and 25(OH)D concentration are scarce. In the present study, dietary vitamin D intake, including intake from food and liquid cod-liver oil, was one of the strongest contributors to satisfactory vitamin D status. This finding is consistent with a previous study on predictors of satisfactory vitamin D status among Norwegian women^(^^4^^)^. In our study, the median of total intake of vitamin D was 11·6 µg/d in males and 9·2 µg/d in females. Daily vitamin D intake was in line with data from a recent national nutritional survey in Norway (Norkost 3)^(^^42^^)^, which reported that mean dietary intake of vitamin D increased from 7 to 12 µg/d in males, and from 5 to 10 µg/d in females, when supplements were included in the calculation. Recently in the Nordic countries, authorities have recommended an increase in vitamin D intake, from 7·5 µg (300IU) to 10 µg (400IU) per day for adults. However, for individuals aged 75 years or older and for those with no or little sun exposure, 20 µg/d (800IU/d) is recommended^(^^43^^)^. In Norway, some types of milk (0·4–0·8 µg vitamin D per 100 g) and all margarine, butter and butter mixed with oil (8 µg vitamin D per 100 g) are enriched with vitamin D^(^^44^^,^^45^^)^.

In our study, smoking and BMI were each inversely associated with S-25(OH)D, and BMI had somewhat greater impact on 25(OH) concentration. A recent systematic review on the non-skeletal health effects of vitamin D supplementation concluded that it is generally agreed that obesity results in low 25(OH)D. However, vitamin D supplementation was not associated with weight loss in randomized clinical trials^(^^46^^)^. The negative association between smoking status (never *v*. current) and 25(OH)D has been consistently described in observational studies^(^^47^^–^^50^^)^. A Danish study found that, in addition to reduced concentration of 25(OH)D, smokers had reduced concentrations of the biologically active form of vitamin D, 1,25-dihydroxyvitamin D, and parathyroid hormone. The study hypothesized that the depression of the vitamin D–parathyroid hormone system seen among smokers is likely to be involved in these associations^(^^51^^)^.

In females, higher alcohol intake (non-consumers *v*.≥6 g alcohol/d) was associated with higher S-25(OH)D. Females reported significantly less alcohol consumption and there were more non-consumers of alcohol among females than males. The association between alcohol consumption and vitamin D concentrations has been previously reported^(^^13^^,^^52^^,^^53^^)^. It has been speculated that alcohol can suppress the conversion of 25(OH)D to 1,25-dihydroxyvitamin D^(^^54^^)^ and, due to that, higher 25(OH)D concentrations might be associated with higher alcohol consumption.

UVB-induced vitamin D production is absent/limited for a considerable part of the year in northern Norway. The number of self-reported daylight hours outside in the week prior to blood sampling was a positive predictor of S-25(OH)D concentration in univariable regression models in males and females. However, this association was not significant in multivariable regression models. In fact, the predictive power of having been on a sunbathing holiday in the past month was much stronger.

The present study has limitations. The response rate was low (47 %) and the generalizability of our results due to selection bias can be questioned. In addition, the sample was limited to individuals aged 40–69 years and cannot be generalized to younger or older individuals. Only ten municipalities were included in the study and geographic comparisons are difficult, as different municipalities were measured at different times of the year. We studied both males and females, but the response was higher among females. Another relevant limitation is that the FFQ has not been specifically validated in males or in the Indigenous Sami population. In addition, we used self-reported data on dietary intake and lifestyle factors, thus we cannot exclude some recall bias. Moreover, it is difficult to accurately measure UVB exposure in large population studies, and we did not have information on protective sun behaviour which could affect vitamin D status. Our study was not designed to investigate the influence of genetic polymorphisms; however, genetic factors have recently been considered to contribute greatly to vitamin D status^(^^47^^,^^55^^–^^57^^)^.

The strength of the present study is the large sample size. Only a few large studies have been designed to simultaneously explore the impact of dietary intakes, supplements, some important factors that related to UVB exposure, and a broad range of lifestyle and sociodemographic factors that are known to influence circulating 25(OH)D concentration. In the present study, trained local medical staff examined participants according to a standard protocol. We used a validated, comprehensive FFQ to estimate total dietary vitamin D^(^^30^^,^^31^^)^, and BMI was calculated based on anthropometric measurements, which are more accurate than self-reported data. We excluded non-Western migrants as they might have inferior 25(OH)D status due to different nutritional and cultural habits^(^^58^^)^.

## Conclusion

In conclusion, our data provide important information for both public health authorities and clinicians. Overall, a quarter of middle-aged residents of rural northern Norway had insufficient vitamin D status on average in the year, although the sunniest summer months were not included. The prevalence of vitamin D deficiency and insufficiency we found was not as high as that in some European and Arctic Indigenous populations, with very few individuals having S-25(OH)D concentration <30 nmol/l. It appears that the Indigenous Sami group has sufficient S-25(OH)D concentrations. Total dietary vitamin D intake was one of the main contributors to higher S-25(OH)D concentration in this multi-ethnic population. In addition, travelling to southern countries for sunbathing vacations can increase these concentrations substantially. Adequate dietary intake of vitamin D is necessary to prevent hypovitaminosis D in this geographical area. Daily intake of vitamin D supplements, preferably cod-liver oil at least during the dark periods (autumn and winter months), as well as a diet rich in vitamin D, seems to be a good prophylactic measure against hypovitaminosis D.

## Supplementary material

For supplementary material accompanying this paper visit https://doi.org/10.1017/S1368980018003816. click here to view supplementary material
