# Supplementary material for: Vitamin D status in a multi-ethnic population of northern Norway: the SAMINOR 2 Clinical Survey
Source: Public Health Nutr. 2019 Feb 15;23(7):1186–200. doi: 10.1017/S1368980018003816 (PMC7167379; doi:10.1017/S1368980018003816)
Supplement: Supplementary file 1 [file S1368980018003816sup.zip › S1368980018003816sup001.docx]

**Supplemental Table S1 Mean (SD) and median S-25(OH)D nmol/L in ethnic groups by season of blood draw and age groups**

|  | **Non-Sami males and females** | | | | **Multiethnic Sami males and females** | | | | **Sami males and females** | | | |
| --- | --- | --- | --- | --- | --- | --- | --- | --- | --- | --- | --- | --- |
|  |  | S-25(OH)D nmol/L | | |  | S-25(OH)D nmol/L | | |  | S-25(OH)D nmol/L | | |
| **Season of blood draw** | n | Mean | SD | Median | n | Mean | SD | Median | n | Mean | SD | Median |
| Nov, Jan.-Apr. | 803 | 62.7 | 20.4 | 60.8 | 358 | 60.3 | 18.3 | 59.7 | 1004 | 61.7 | 18.1 | 60.3 |
| May-Jun. | 821 | 63.4 | 19.7 | 62.0 | 108 | 63.9 | 19.9 | 60.3 | 56 | 62.6 | 19.9 | 59.4 |
| Sep.-Oct. | 1094 | 68.1 | 18.5 | 66.4 | 151 | 68.5 | 18.6 | 64.7 | 70 | 63.8 | 20.5 | 64.0 |
| **Age groups** |  |  |  |  |  |  |  |  |  |  |  |  |
| 40-49 | 728 | 59.1 | 18.5 | 56.7 | 204 | 55.3 | 17.4 | 53.6 | 269 | 56.2 | 17.4 | 53.2 |
| 50-59 | 862 | 66.1 | 19.3 | 64.6 | 202 | 63.8 | 17.1 | 63.3 | 389 | 60.8 | 18.2 | 58.2 |
| 60-69 | 1128 | 68.2 | 19.6 | 66.5 | 211 | 69.4 | 19.5 | 66.8 | 472 | 66.0 | 18.0 | 65.3 |

SD: standard deviation; S-25(OH)D: serum 25-hydroxyvitamin D
