# Supplementary material for: Vitamin D status in a multi-ethnic population of northern Norway: the SAMINOR 2 Clinical Survey
Source: Public Health Nutr. 2019 Feb 15;23(7):1186–200. doi: 10.1017/S1368980018003816 (PMC7167379; doi:10.1017/S1368980018003816)
Supplement: Supplementary file 1 [file S1368980018003816sup.zip › S1368980018003816sup002.docx]

**Supplemental Table S2 The relative importance of predictors in males and females using dominance analysis**

|  | Males | | Females | |
| --- | --- | --- | --- | --- |
| Variable | Contribution to R^2^ (%) | Rank | Contribution to R^2^ (%) | Rank |
| Age | 11.00 | 3 | 19.63 | 2 |
| Smoking status | 0.61 | 9 | 2.45 | 10 |
| BMI | 5.71 | 6 | 3.56 | 7 |
| Alcohol | / | / | 2.99 | 9 |
| Season of the blood collection | 6.91 | 5 | 5.07 | 6 |
| Sun bathing holiday in the past month | 29.41 | 1 | 15.18 | 3 |
| Solarium | 4.68 | 7 | 6.71 | 5 |
| Use of vitamin/mineral supplements | 10.02 | 4 | 13.02 | 4 |
| Cod liver oil capsules/fish oil capsules | 3.16 | 8 | 3.12 | 8 |
| Vitamin D intake (µg / per day) † | 28.51 | 2 | 28.28 | 1 |
| R^2^ | 0.21 | | 0.22 | |

† Vitamin D intake (continuous)
